# Diazoxide Post-conditioning Activates the HIF-1/HRE Pathway to Induce Myocardial Protection in Hypoxic/Reoxygenated Cardiomyocytes

**DOI:** 10.3389/fcvm.2021.711465

**Published:** 2021-12-06

**Authors:** Xi-Yuan Chen, Jia-Qi Wang, Si-Jing Cheng, Yan Wang, Meng-Yuan Deng, Tian Yu, Hai-Ying Wang, Wen-Jing Zhou

**Affiliations:** ^1^Department of Anesthesiology, The Affiliated Hospital of Zunyi Medical University, Guizhou, China; ^2^Department of Anesthesiology, The Xinqiao Hospital of Army Medical University, Chongqing, China; ^3^Guizhou Key Laboratory of Anesthesia and Organ Protection, Affiliated Hospital of Zunyi Medical University, Guizhou, China

**Keywords:** hypoxic reoxygenation injury, diazoxide, myocardial protection, HIF-1/HRE pathway, cardiomyocytes

## Abstract

**Background:** Previous studies have shown that diazoxide can protect against myocardial ischemia-reperfusion injury (MIRI). The intranuclear hypoxia-inducible factor-1 (HIF-1)/hypoxia-response element (HRE) pathway has been shown to withstand cellular damage caused by MIRI. It remains unclear whether diazoxide post-conditioning is correlated with the HIF-1/HRE pathway in protective effect on cardiomyocytes.

**Methods:** An isolated cardiomyocyte model of hypoxia-reoxygenation injury was established. Prior to reoxygenation, cardiomyocytes underwent post-conditioning treatment by diazoxide, and 5-hydroxydecanoate (5-HD), N-(2-mercaptopropionyl)-glycine (MPG), or dimethyloxallyl glycine (DMOG) followed by diazoxide. At the end of reoxygenation, ultrastructural morphology; mitochondrial membrane potential; interleukin-6 (IL-6), tumor necrosis factor alpha (TNF-α), reactive oxygen species (ROS), and HIF-1α levels; and downstream gene mRNA and protein levels were analyzed to elucidate the protective mechanism of diazoxide post-conditioning.

**Results:** Diazoxide post-conditioning enabled activation of the HIF-1/HRE pathway to induce myocardial protection. When the mitoK_ATP_ channel was inhibited and ROS cleared, the diazoxide effect was eliminated. DMOG was able to reverse the effect of ROS absence to restore the diazoxide effect. MitoK_ATP_ and ROS in the early reoxygenation phase were key to activation of the HIF-1/HRE pathway.

**Conclusion:** Diazoxide post-conditioning promotes opening of the mitoK_ATP_ channel to generate a moderate ROS level that activates the HIF-1/HRE pathway and subsequently induces myocardial protection.

## Introduction

Myocardial ischemic-reperfusion injury (MIRI) is the major cause of increased post-operative infarction rate and mortality, especially given the increasing numbers of cardiac procedures performed, including heart transplantation, cardiopulmonary bypass surgery, coronary stent placement, and thrombolytic procedures (1). Hypoxia-inducible factor-1 (HIF-1) is reported to be a crucial component in the adaptation of cell physiology, controlling vascularization, regulating oxidative stress, and affecting proliferation and apoptosis (2, 3). HIF-1 is a heterodimer composed of the rate-limiting factor, HIF-1α, and the constitutively expressed HIF-1β. HIF-1α is induced by hypoxia as well as by oncogenes and pharmacological intervention. During normoxia, the unstable HIF-1α protein is rapidly degradable by the proteasome resulting from its ubiquitination by the product of the Von Hippel Lindau tumor suppressor gene (VHL). However, HIF-1α cannot be hydroxylated during hypoxia. Under these circumstances, HIF-1α accumulates and binds to HIF-1β to form the active transcription factor HIF-1. The HIF-1 complex can bind to the hypoxia-response element (HRE) sequences in the promoter of HIF-1 target genes to activate the HIF-1/HRE pathway, which itself participates in hypoxic adaptation (4). HIF-1 has a role in preconditioning and post-conditioning to withstand cell damage caused by MIRI (5, 6). *In vivo* and isolated MIRI model studies have shown that diazoxide can maintain cellular functions to alleviate MIRI (7). Therefore, we examined whether protection by diazoxide post-conditioning was related to the HIF-1/HRE signaling pathway.

Reactive oxygen species (ROS) describe O^2−^, H_2_O_2_, NO, and –OH radicals and other single-electron reduction products that are directly generated by the mitochondrial electron transport chain when mitoK_ATP_ channels are opened (8). Several complex intrinsic signaling pathways are known to play a key role in the protective mechanism of the myocardium by ultimately affecting ROS. Studies have confirmed that ROS are important stimulating factors of the myocardium at the molecular level, and can be used as a protective signal-mediated molecule to stimulate endogenous signal pathways during the ischemia and reperfusion period. However, it remains unknown whether diazoxide can induce myocardial protection by regulating the ROS level during ischemia and reperfusion.

Therefore, we established an isolated cardiomyocyte model of hypoxic/reoxygenation injury to observe whether the protective effect of diazoxide post-conditioning is related to the HIF-1/HRE pathway. In addition, we also examined the internal connection between the HIF-1/HRE pathway and ROS in diazoxide post-conditioning.

## Materials and Methods

### Drugs and Reagents

Diazoxide, mitoK_ATP_ blocker −5-hydroxydecanoate (5-HD), ROS scavenger—N-2-mercaptopropionylglycine (MPG), and HIF-1 agonist—dimethyloxaloylglycine (DMOG) were purchased from Sigma (St. Louis, MO, USA). Rabbit-anti-HIF-1α monoclonal antibody, rabbit-anti VEGF monoclonal antibody, and rabbit-anti-GAPDH monoclonal antibody were purchased from Sigma. Pentobarbital was purchased from Shanghai Tyrael Biological Technology Co., Ltd. The study was approved by the Institutional Review Board of The Affiliated Hospital of Zunyi Medical University, Guizhou, China.

### Establishment of the Hypoxic/Reoxygenation Model

The experimental animals used were 90 healthy male Sprague Dawley (SD) rats (weight, 250–300 g; 16–20 weeks old). Each rat was injected intraperitoneally with 250 U/kg heparin for anticoagulation, as well as 40 mg/kg sodium pentobarbital for anesthesia. After cutting though the rib cage, the heart was immediately isolated and placed in a Ca^2+^ solution (130 mmol/L NaCl, 5.4 mmol/L KCl, 3.5 mmol/L MgCl_2_·6H_2_O, 0.4 mmol/L NaH_2_PO_4_, 10 mmol/L glucose, 5 mmol/L HEPES, 750 μmol/L CaCl_2_, pH 7.25–7.35) at 4°C to drain the blood. The heart was then fixed onto a MAP perfusion needle, and the aorta was retrogradely perfused with an oxygenated (95% air, 5% CO_2_, 37°C) Ca^2+^ solution of 9 mL/min/g for 3 min. The ventricle was removed after perfusion with EGTA solution (130 mmol/L NaCl, 5.4 mmol/L KCl, 3.5 mmol/L MgCl_2_·6H_2_O, 0.4 mmol/L NaH_2_PO_4_, 10 mmol/L glucose, 5 mmol/L HEPES, 100 μmol/L EGTA, pH 7.25–7.35) for 4 min and Type II collagenase solution (130 mmol/L NaCl, 5.4 mmol/L KCl, 3.5 mmol/L MgCl_2_·6H_2_O, 0.4 mmol/L NaH_2_PO_4_, 10 mmol/L glucose, 5 mmol/L HEPES, 90 μmol/L Cl_2_, 0.1% Type II collagenase, pH 7.25–7.35) for 8–10 min. Then, the ventricle was immersed in 3 mL Type II collagenase solution and placed in a horizontal shaker at 37°C to separate the cardiomyocytes into the solution. The cell suspensions were filtered through a 200-μm mesh gauze into a sterile tube to eliminate superfluous cellular material. After 5 min, the intact active myocytes were found precipitated to the bottom of the tube. Myocyte precipitates were washed with enzyme eluent (130 mmol/L NaCl, 5.4 mmol/L KCl, 3.5 mmol/L MgCl_2_·6H_2_O, 0.4 mmol/L NaH_2_PO_4_, 10 mmol/L glucose, 5 mmol/L HEPES, 1% BSA, pH 7.25–7.35) thrice. Then, single myocytes were gently resuspended in M199 solution at 37°C. Cells were observed under a microscope to evaluate cell survival rate. When cell survival rate was >90%, downstream experiments could be performed.

After separation, filtration, and dispersion, cardiomyocyte suspensions were plated on cell culture dishes. Six-well culture dishes were coated with 800–1,000 μL of laminin (10 μg/mL, L2020-1MG, Sigma-Aldrich) per well and allowed to dry for 30 min. The cardiomyocyte suspension was then added to each well at a final density of 10^4^-10^6^/cm^2^. The cells could adhere to the culture dishes after 4 h at 37°C. Then, cell-culture medium and unattached cells were removed from the culture dishes and 2 mL of M199 solution was pipetted into each well. After refreshing the cell culture medium, cardiomyocytes were incubated for 20 h at 37°C with 95% air and 5% CO_2_ (9, 10).

### Experimental Groups

The cardiomyocytes were randomly divided into the following six experimental groups: sham group (N); control group (hypoxia-reoxygenation group, H/R); diazoxide post-conditioning group (D); 5-HD (mitoK_ATP_ blocker) +diazoxide group (5-HD+D); MPG (ROS scavenger) +diazoxide group (MPG+D), and ROS scavenger+HIF1 stabilizer+diazoxide group (MPG+DMOG+D).

**Figure 1:**
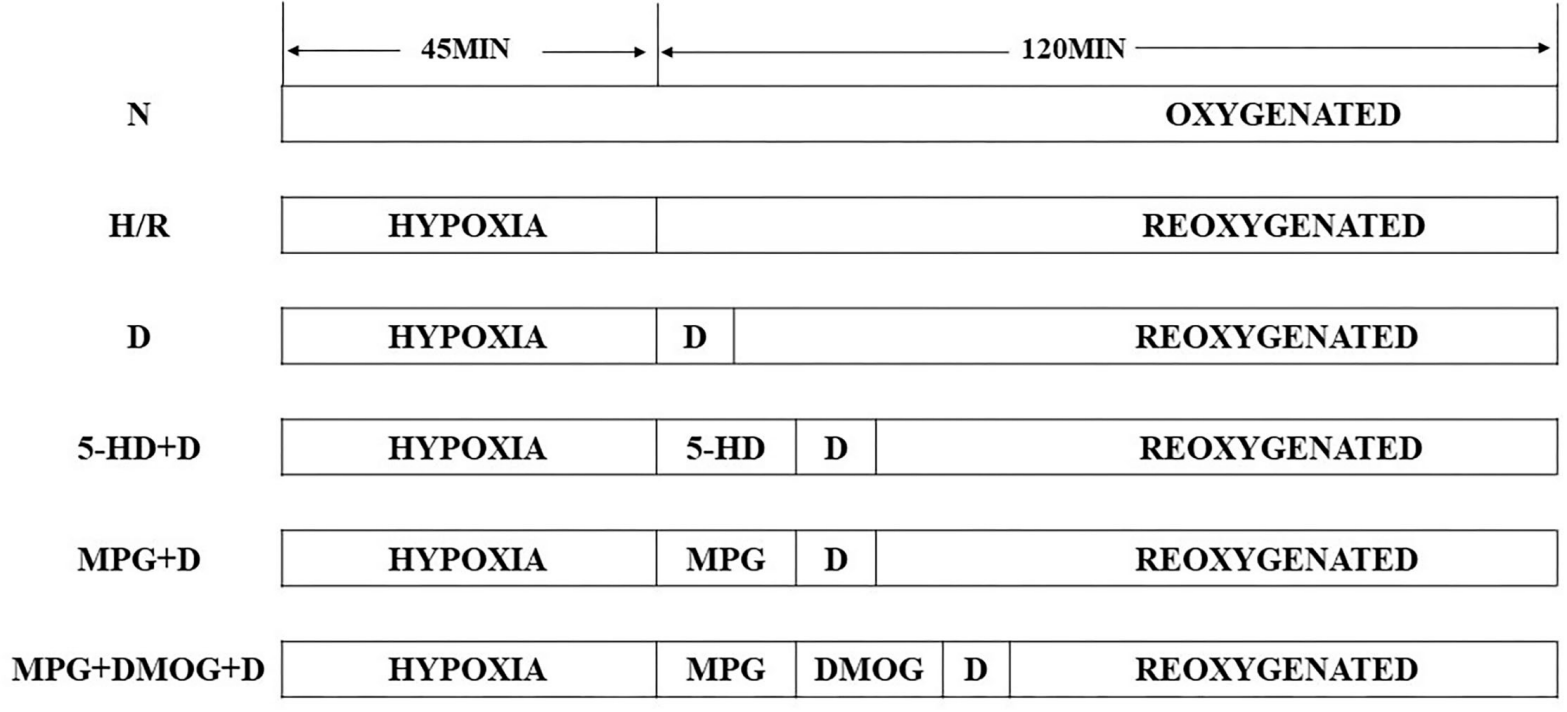
Schematic representation.

The cardiomyocytes in the sham group were persistently cultured in an incubator under normal oxygen conditions (95% air+5% CO_2_) for 165 min. Those in the H/R group were subjected to hypoxia (5% CO_2+_ 1% O_2+_ 94% N_2_) for 45 min, then reoxygenated (95% air+5% CO_2_) for 120 min. The D group cardiomyocytes were first subjected to hypoxia for 45 min, then subjected to 3 min of diazoxide (50 μmol/L) post-conditioning, followed by reoxygenation for 117 min. The cardiomyocytes in the 5-HD+D group were subjected to hypoxia for 45 min, 5-HD (100 μmol/L) treatment for 10 min, diazoxide (50 μmol/L) post-conditioning for 3 min, followed by reoxygenation for 107 min in turn. The cardiomyocytes in the MPG+D group were subjected to hypoxia for 45 min, MPG (2 mmol/L) for 10 min, diazoxide (50 μmol/L) post-conditioning for 3 min, followed by reoxygenation for 107 min in turn. On the basis of these treatments, the cardiomyocytes in the MPG+DMOG+D group were subjected to hypoxia for 45 min, MPG (2 mmol/L) for 10 min, DMOG (100 μmol/L) for 10 min, diazoxide (50 μmol/L) post-conditioning for 3 min, followed by reoxygenation for 97 min in turn (Figure 1).

### Observation of the Changes in Myocardial Ultrastructure and Mitochondrial Flameng Score

After reoxygenation, the cardiomyocytes were collected from the dishes. The cardiomyocytes were first centrifuged in medium (225 g, 10 min, 4°C, 5804R, Eppendorf, Germany) to form the cell pellet. Then, the cardiomyocytes were fixed with 4°C glutaraldehyde phosphate buffer before being subjected to gradient dehydration, washing, embedding, and permeation. They were cut into ultrathin 60-nm slices, and the slices were stained with uranyl acetate (Sigma-Aldrich) for 15 min, followed by lead citrate (Solarbio, Beijing, China) for 20 min. The myocardial ultrastructure and Flameng scoring of the mitochondria were performed using an H7500 transmission electron microscope (×10,000; Hitachi, Tokyo, Japan). A total of 100 mitochondria were randomly selected from each slice, and the average score of the 100 mitochondria represented the sample score. The mean±SD of the mitochondrial scores for each sample was calculated. In brief, mitochondrial scores for the respective samples were determined to assign a score from 0 to 4, with higher scores indicating more injury, as follows: normal mitochondrial structure and intact particles, 0 points; normal mitochondrial structure but missing particles, 1 point; mitochondrial swelling and transparent matrix, 2 points; considerable rupture and transparent, concentrated matrix, 3 points; rupture, with incomplete inner and outer mitochondrial membranes, 4 points (11).

### Measurement of Mitochondrial Membrane Potential

Adherent cells were grown in a standard petri dish and treated according to their group as described in text above. After reoxygenation, the seeded cells were washed with medium once and 1 mL/well JC-1 working solution was added and incubated for 20 min at 37°C in the dark. After two washings with 1 × dilution buffer (1 ml of JC-1 5X and 4 ml of distilled water), the cells could be observed on a fluorescent microplate reader. JC-1 is a cationic dye that accumulates in energized mitochondria. At low mitochondrial membrane potential, JC-1 is predominantly a monomer that yields green fluorescence with emission of 530 ± 15 nm. At high mitochondrial membrane potential, the dye aggregates and yields a reddish orange emission (590 ± 17.5 nm). Data obtained with the JC-1 assay provides a relative measure of mitochondrial membrane potential by a percentage of red and green fluorescence. Therefore, a decrease in the aggregate fluorescent count is indicative of depolarization, whereas an increase is indicative of hyperpolarization.

### Measurement of IL-6 and TNF-α Content

At the end of reoxygenation, adherent cardiomyocytes were collected and centrifuged in medium (225 g, 10 min, 4°C, 5804R, Eppendorf, Germany). The cardiomyocyte sedimentation was crushed ultrasonically in 1 mL of PBS. Lactate, IL-6, and TNF-α levels were measured using assay kits (IL-6 ELISA kit PI328, TNF-α ELISA kit PT512, Beyotime Biotechnology, Jiangsu, China) according to the manufacturer's instructions.

### Quantitative Real-Time Polymerase Chain Reaction

To evaluate whether the HIF-1/HRE pathway was activated and the function it served, we measured transcripts of HIF-1α and its downstream genes. At the end of reoxygenation, total RNA from cardiomyocytes was extracted with TRIzol using an RNA extraction kit (TaKaRa, Dalian, China), according to the manufacturer's instructions. The concentration and purity of the total RNA was determined by spectrophotometry at 260 and 280 nm, with an OD_260_/OD_280_ ratio between 1.8 and 2.0 suggesting that the total RNA was pure. Complementary DNA (cDNA) was synthesized from total RNA using the QuantiTect Reverse Transcription kit (TaKaRa). The mRNA levels of HIF-1α, VEGF, Bcl-2, and Bax were measured by RT-PCR, and the following specific primers were used (TaKaRa):

HIF-1α, forward: GGTGCTAACAGATGATGGTGAC,
reverse: GGCTCATAACCCATCAACT.
VEGF, forward: CAATGATGAAGCCCTGGAGTG,
reverse: GTCTGCGGATCTTGGACAAAC
Bcl-2, forward: TGGATGACTGAGTACCTGAA,
reverse: GGCCTACTGACTTCACTTAT.
Bax, forward: AGGAGCAGCTGCTTGTCT,
reverse: GGTCCCGAAGTAGGAGAGGA.

### Western Blotting

At the end of reoxygenation, adherent cardiomyocytes were washed twice with PBS and 1 mL of strong RIPA cell lysate (strong RIPA cell lysate: PMSF, 100:1), then centrifuged (12,000 *g* at 4°C) for 5 min. The supernatant was aliquoted, and protein content was determined using the Micro BCA protein assay kit (Beyotime). After denaturation at 100°C, proteins were subjected to 10% sodium dodecyl sulfate-polyacrylamide gel electrophoresis (SDS-PAGE) (Amresco, Solon, OH, USA). The fractionated products were electrophoretically transferred onto a polyvinylidene difluoride membrane (Hybond-P, Bio-Rad, Berkeley, CA, USA) and blocked at 37°C for 2 h. The membrane was then incubated overnight with primary antibodies. HIF-1α, VEGF, Bcl-2, and Bax were probed with 1:1,000 dilutions of the respective antibodies overnight at 4°C. The membrane was then washed with TBST buffer and incubated with secondary antibodies (1:10,000) at room temperature for 2 h. The fluorescence intensity of each membrane was measured by an infrared fluorescent imaging system (NatureGene Corp, Beijing, China) to determine the fluorescence intensity of the target membrane.

### Measurement of Reactive Oxygen Species (ROS) Content in Cardiomyocytes

At 25 min and 120 min of reoxygenation, the cardiomyocytes were separately collected and centrifuged in medium (225 g, 10 min, 4°C, 5804R, Eppendorf, Germany), and finally made into cell lysate using a 50 μL RIPA lysate. The ROS content of each group cell lysate was quantified using an ROS assay kit (JL21051, Beyotime Biotechnology).

### Statistical Analysis

Graphpad Prism 7.0 (GraphPad Software, La Jolla, CA) were used for statistical analysis. Two-way ANOVA followed by Sidak's multiple comparisons test was used to compare ROS concentration between multiple groups at the different time point. Flameng score were analyzed by conducting Kruskal-Wallis test followed by Dunn's multiple comparisons test, whereas all other dataset were considered normally distributed and therefore analyzed by one-way ANOVA followed by Tukey's multiple comparisons tests. Quantitative data were expressed as the mean ± standard deviation. *P* < 0.05 was considered to indicate statistical significance.

## Results

**1. Diazoxide post-conditioning activates the HIF-1/HRE pathway by regulating mitoK**_**ATP**_ **and ROS**.

### Cardiomyocyte Ultrastructure

**Figure 2:**
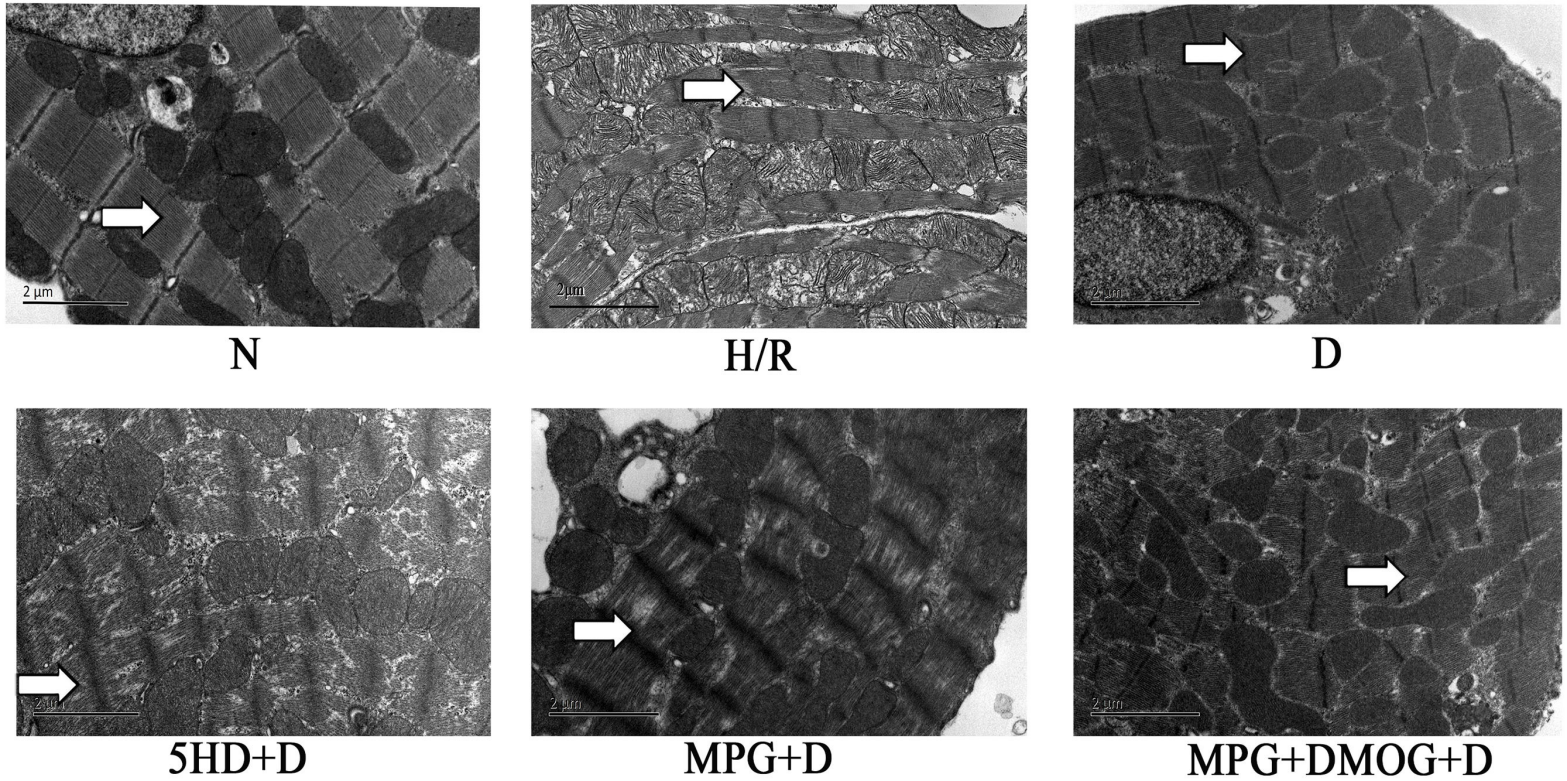
Myocardial ultrastructure. At the end of reoxygenation, cardiomyocytes were examined under an electron microscope (magnification: 10,000×).

The ultrastructures of myofilaments and organelles were observed using electron microscopy. In the normal group (N), the myofilaments and organelles were appropriately arranged, intact, and clearly distinguishable. The myofilaments in the H/R group were ruptured, dissolved, or even severed, and prominent organelle swelling was observed, suggesting incomplete mitochondria. The D group was protected compared with the H/R group, as the cardiomyocytes were intact with mitochondria-rich myofilaments, and the filaments were focally dissolved with clear bright and dark bands and an evident Z-line. The 5-HD+D and MPG+D groups exhibited some impairments such as ruptured myofilaments arrangement, as well as mitochondrial cristae swelling and breakage. The MPG+DMOG+D group showed comparable results to the D group (Figure 2).

### Mitochondrial Flameng Score

**Figure 3:**
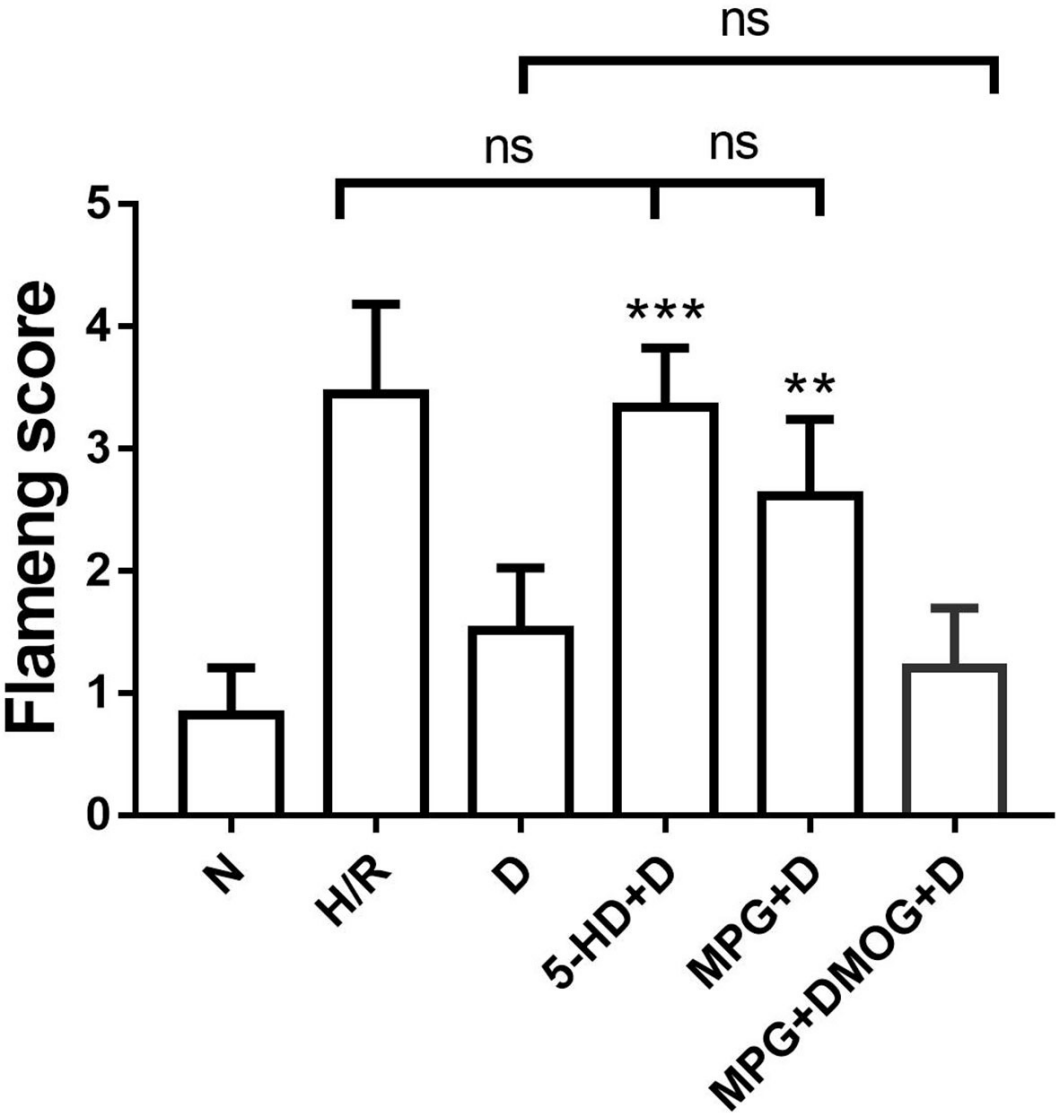
Mitochondrial Flameng score. ^**^P < 0.01 vs. D group; ^***^P < 0.001 vs. D group. *N* = 6/group. Each value is the result of 6 experiments performed with different isolated preparations of cardiomyocytes. Flameng scores were analyzed by Kruskal-Wallis test followed by Dunn's multiple comparisons test.

There were appreciable differences in the Flameng score between the N group and the H/R group. However, the Flameng score of the D group was significantly decreased compared with that of the H/R group (*P* < 0.001). Nonetheless, the Flameng score was increased in the 5-HD+D group (*P* < 0.001) and the MPG+D group (*P* = 0.003) compared with the D group. In addition, the Flameng score of the MPG+DMOG+D group was similar to that of the D group (*P* > 0.999) (Figure 3).

### Mitochondrial Membrane Potential

**Figure 4:**
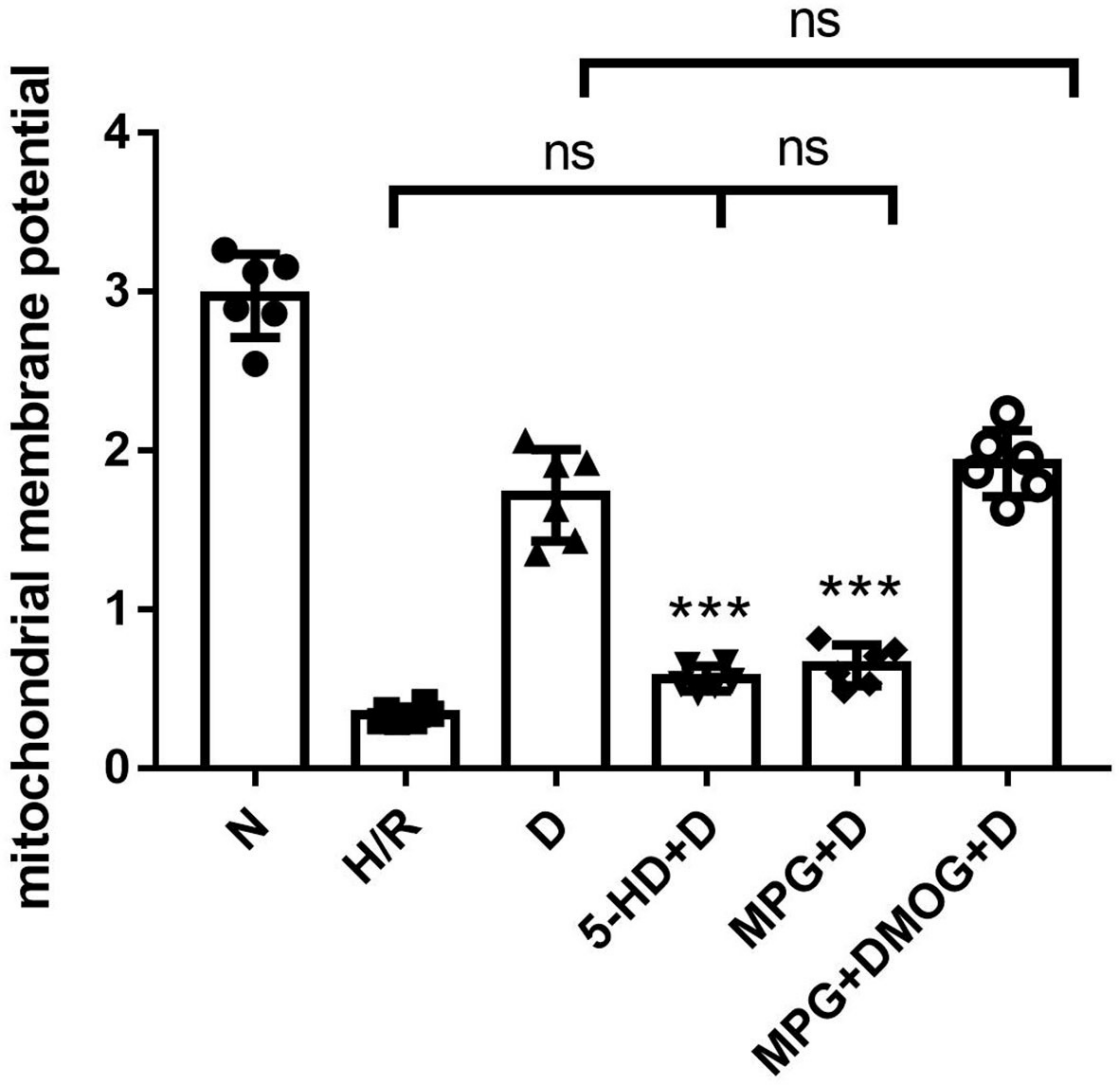
Mitochondrial membrane potential. ^***^P < 0.001 vs. D group. *n* = 6/group. Each value is the result of 6 experiments performed with different isolated preparations of cardiomyocytes. One-way ANOVA followed by Tukey's multiple comparisons tests was used for analyze.

Diazoxide post-conditioning improved the mitochondrial membrane potential in the D group. However, the protective effects of diazoxide on mitochondrial membrane potential were reduced by administration of 5-HD (*P* < 0.001) and MPG (*P* < 0.001). The administration of DMOG in the MPG+DMOG+D group could show a protective effect compared with the MPG+D group (*P* < 0.001) (Figure 4).

### IL-6 and TNF-α Content

**Figure 5:**
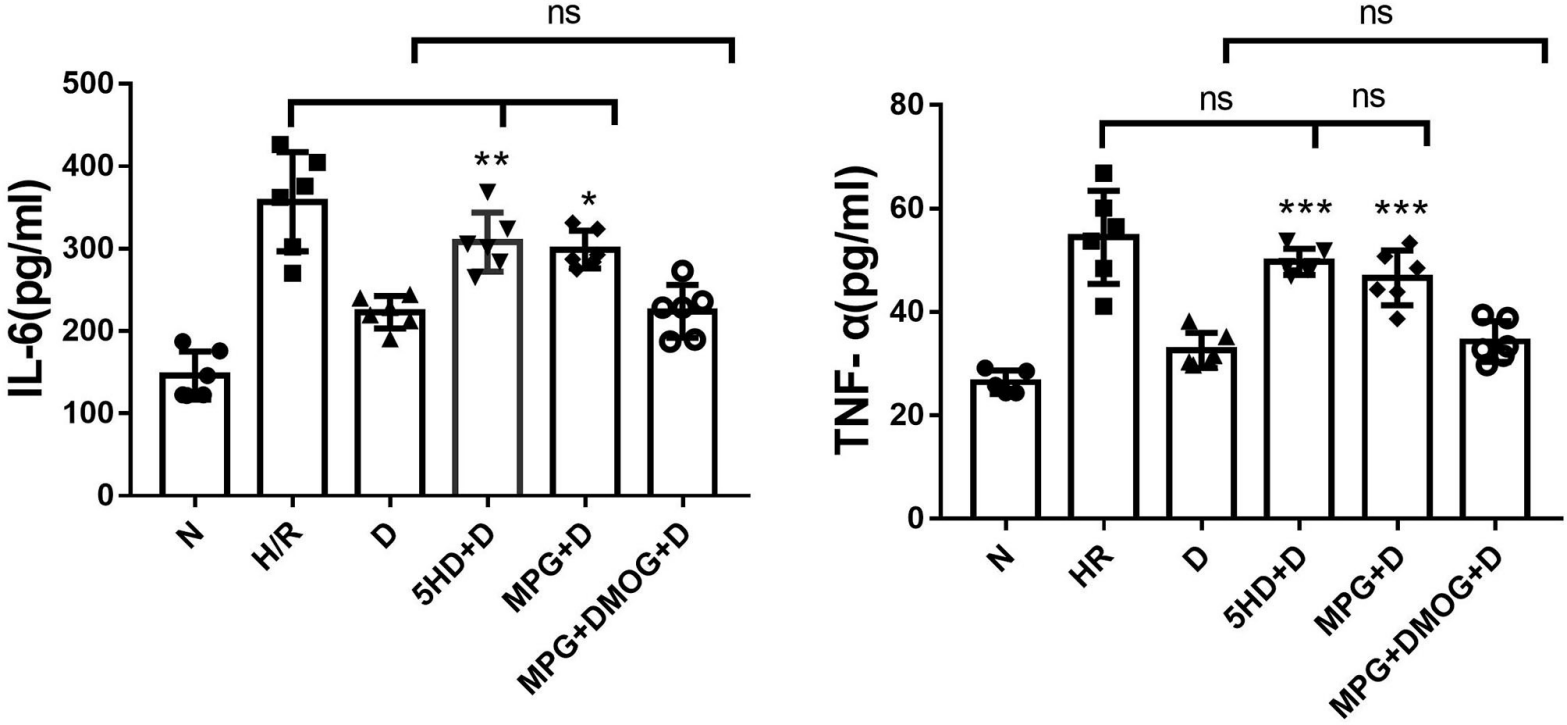
IL-6 and TNF-α content. ^*^P < 0.05 vs. D group; ^**^P < 0.01 vs. D group; ^***^P < 0.001 vs. D group. *n* = 6/group. Each value is the result of 6 experiments performed with different isolated preparations of cardiomyocytes. One-way ANOVA followed by Tukey's multiple comparisons tests was used for analyze.

IL-6 and TNF-α were markedly decreased in the D group compared with the H/R group (*P* < 0.001). However, the protective effects of diazoxide on IL-6 and TNF-α were eliminated by administration of 5-HD and MPG, while in the 5-HD+D (*P* < 0.001) and MPG+D groups (*P* = 0.010), IL-6 and TNF-α were increased. Administration of DMOG in the MPG+DMOG+D group reduced IL-6 and TNF-α levels compared with the MPG+D group (*P* = 0.012) (Figure 5).

### Expression of HIF-1α, VEGF, Bcl-2, and Bax mRNA

**Figure 6:**
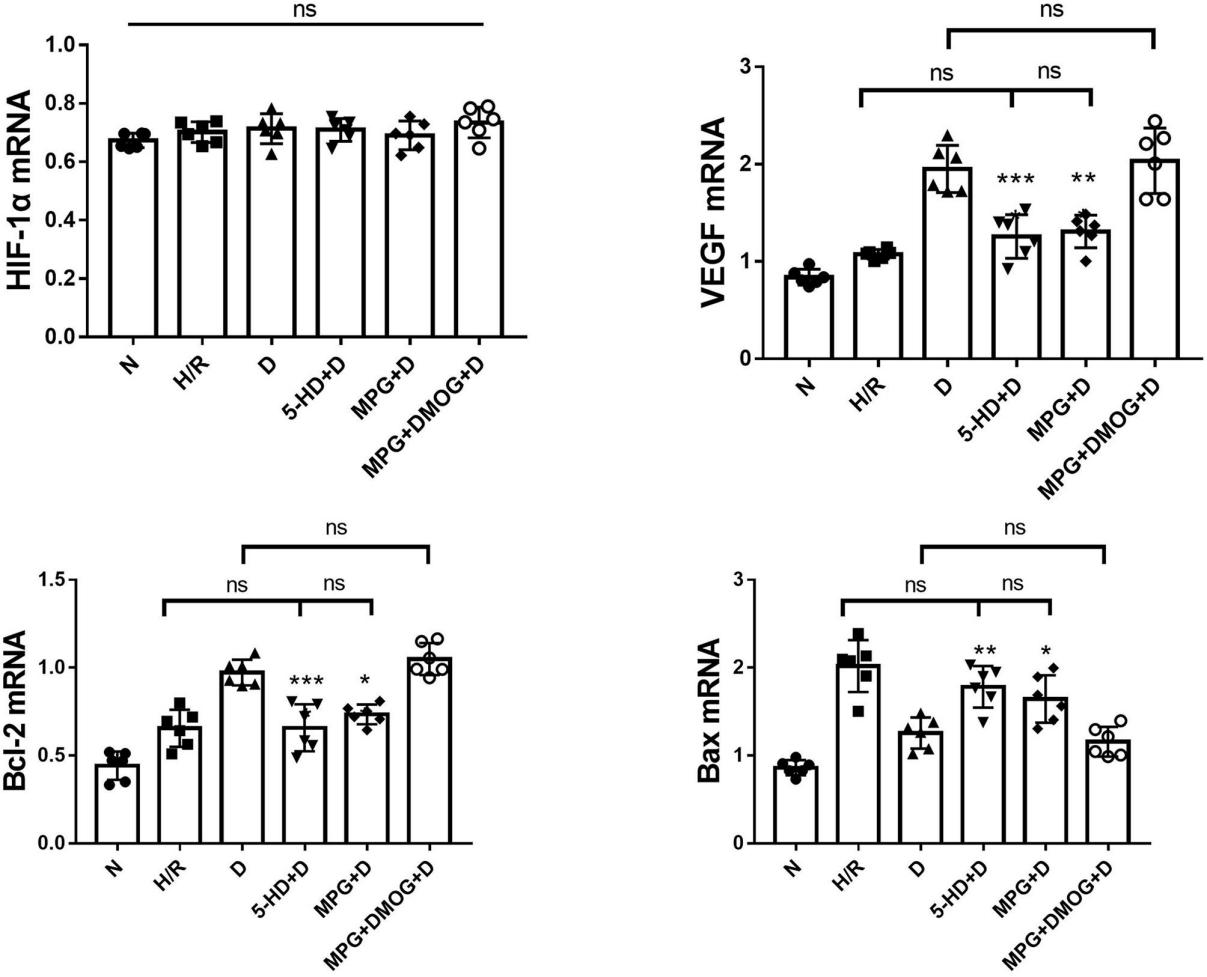
mRNA expression of HIF-1α, VEGF, Bcl-2, and Bax. *P < 0.05 vs. D group; ^**^P < 0.01 vs. D group; ^***^P < 0.001 vs. D group. *n* = 6/group. Each value is the result of 6 experiments performed with different isolated preparations of cardiomyocytes. One-way ANOVA followed by Tukey's multiple comparisons tests was used for analyze.

There were no appreciable differences in HIF-1α mRNA expression among all groups (*P* = 0.168). Diazoxide upregulated the expression of VEGF and Bcl-2 and downregulated the expression of Bax compared with the H/R group (*P* < 0.001). However, 5-HD and MPG treatment diminished the positive effect of diazoxide. The expression of VEGF and Bcl-2 was decreased and the expression of Bax was increased in the 5-HD+D and MPG+D groups to levels similar to those in the H/R group (*P* = 0.387). After DMOG treatment, the expression levels in the MPG+DMOG+D group were similar to those in the D group (*P* = 0.720), potentially counteracting the negative effect of MPG (*P* = 0.007) (Figure 6).

### Expression of HIF-1α, VEGF, Bcl-2, and Bax Proteins

**Figure 7:**
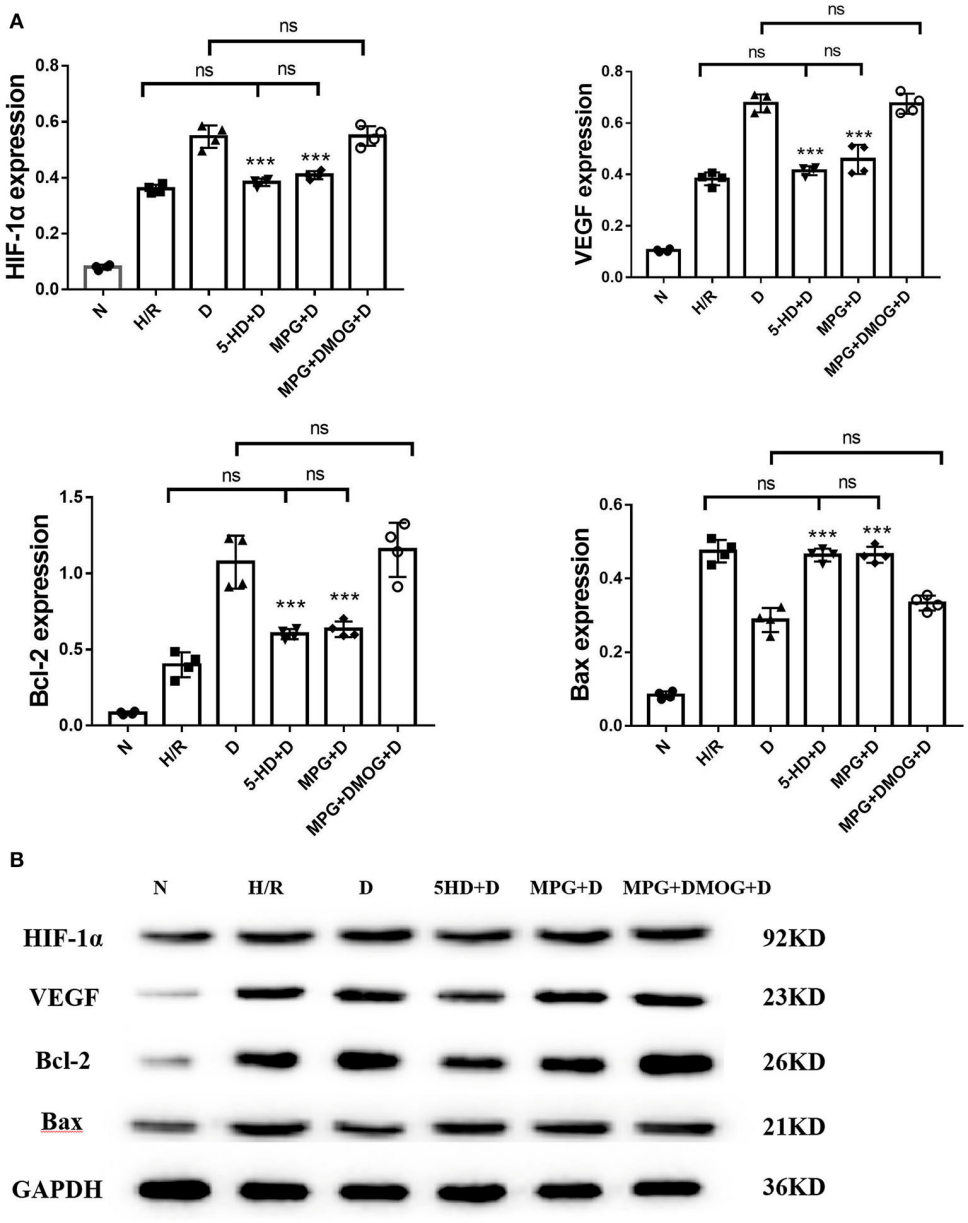
**(A, B)** Protein expression of HIF-1α, VEGF, Bcl-2, and Bax. ^***^P < 0.001 vs. D group. *n* = 4/group. Each value is the result of 4 experiments performed with different isolated preparations of cardiomyocytes. One-way ANOVA followed by Tukey's multiple comparisons tests was used for analyze.

At the end of reoxygenation, the D group clearly showed elevated protein levels of HIF-1α and its downstream mediators, VEGF and Bcl-2, compared with other groups, as well as reduced protein levels of Bax (*P* < 0.001). However, this upregulation was eliminated by 5-HD and MPG treatment. Protein expression levels in the 5-HD+D (*P* = 0.150) and MPG+D groups (*P* = 0.052) were close to those in the H/R group. DMOG treatment restored the effect of diazoxide after MPG treatment, with expression levels in the MPG+DMOG+D group close to those in the D group (*P* = 0.104) (Figure 7).

**2. Although production of a large amount of ROS may cause injury in early reoxygenation, a moderate level of ROS as generated by diazoxide post-conditioning is critical for activation of the HIF-1/HRE pathway**.

ROS concentration following 25 min of reoxygenation was lowest in the N group. In the H/R group, a large amount of ROS was produced in the cells in early reoxygenation. After diazoxide post-conditioning, the early reoxygenation ROS content of the D group was significantly lower than that of the H/R group (*P* < 0.001). Compared with the D group, the 5-HD+D group showed increased concentration (*P* = 0.010), whereas the MPG+D group and MPG+DMOG+D groups showed decreased concentration (*P* < 0.001). There were no significant differences between the MPG+DMOG+D and MPG+D groups (*P* > 0.999).

The ROS concentration at 120 min of reoxygenation was lowest in the N group and highest in the H/R group. The D group showed significantly lower concentration than the H/R group (*P* < 0.001). Compared with the D group, ROS concentration in the 5-HD+D (*P* = 0.001) and MPG+D groups increased (*P* = 0.014). The concentration of the MPG+DMOG+D group was lower than that of the MPG+D group (*P* < 0.001).

**Figure 8:**
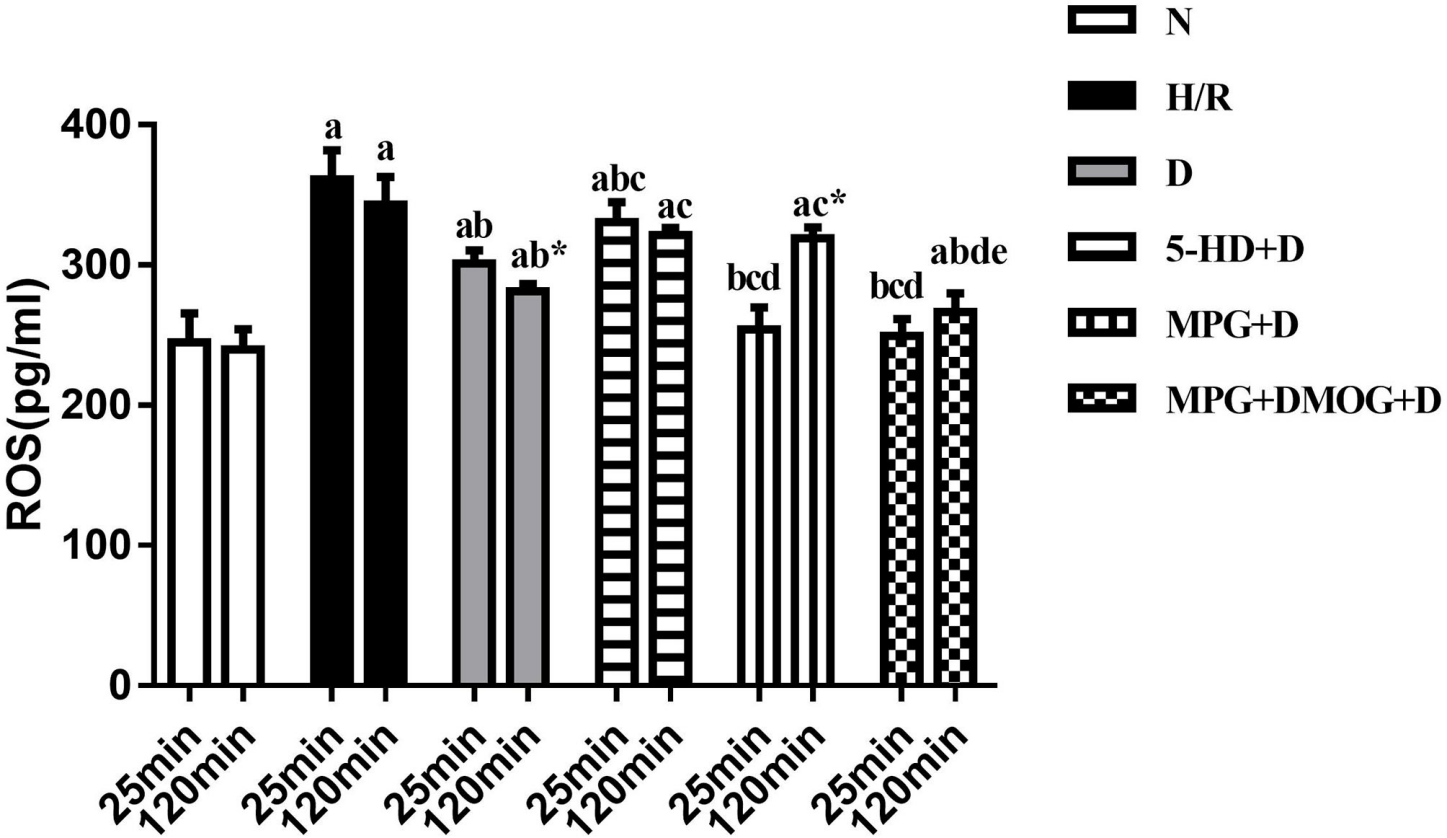
ROS content. ^a^*P* < 0.05 vs. N group; ^b^*P* < 0.05 vs. H/R group; ^c^*P* < 0.05 vs. D group; ^d^*P* < 0.05 vs. 5-HD+D group. ^e^*P* < 0.05 vs. MPG+D group. ^*^P < 0.05 vs. 25 min. *n* = 6/group. Each value is the result of 6 experiments performed with different isolated preparations of cardiomyocytes. Two-way ANOVA followed by Sidak's multiple comparisons test was used to compare ROS concentration between multiple groups at the different time point.

Comparison of ROS concentration at 25 and 120 min of reoxygenation revealed that the ROS content of the cells in the N group continued to be low. In the H/R group, a large amount of ROS was produced in the cells in early reoxygenation, and the level of ROS at the end of reoxygenation was higher. After diazoxide post-conditioning, the D group showed a moderate level of ROS in early reoxygenation, and ROS accumulation after reoxygenation was significantly lower than that in the H/R group (*P* < 0.001). After using the ROS scavenger, MPG, in the early stage of reoxygenation, the accumulation of ROS in the cells was much greater in the MPG+D group than in the D group at the end of reoxygenation (*P* = 0.004). After the addition of DMOG after MPG, DMOG was able to reverse the effect of MPG, and ROS levels at the end of reoxygenation were consistent with those of group D (*P* = 0.855) (Figure 8).

## Discussion

This study demonstrates that under hypoxia-reoxygenation conditions, diazoxide post-conditioning in myocardial protection is associated with regulation of the HIF-1/HRE pathway. 5-HD-induced inhibition of the mitoK_ATP_ channel and MPG elimination of ROS led to the loss of diazoxide post-conditioning in myocardial protection. HIF-1/HRE pathway-dependent diazoxide post-conditioning is related to mitoK_ATP_ channel opening and the level of ROS. However, MPG followed by DMOG treatment was able to reverse the effect of the absence of ROS and activate the HIF-1/HRE signaling, thereby restoring diazoxide post-conditioning-induced myocardial protection. This suggests that ROS produced by the mitoK_ATP_ channel could exert a similar effect to DMOG in order to maintain the stability of HIF-1α. This research further indicates that diazoxide post-conditioning facilitates mitoK_ATP_ channel opening, releasing an appropriate amount of ROS at the early stage of reoxygenation that could be critical to activating HIF-1/HRE pathway.

Mounting evidence has confirmed that the most effective early method of reducing myocardial ischemic injury is revascularization (12). Unfortunately, the reperfusion process can further induce MIRI through a variety of pathophysiological mechanisms (13–16). Ischemic pre-/post-conditioning and pharmacological pre-/post-conditioning as exogenous interventions can activate reperfusion injury salvage kinase (RISK), phosphatidylinositide-3 kinase (PI3K)-AKT, extracellular signal-regulated kinase (ERK)1/2, glycogen synthase kinase (GSK)-3β, and other pathways through endogenous protective mechanisms to produce myocardial protection, and many of these pathways are regulated by HIF-1 (17). HIF-1α is considered the rescue headquarters for ischemia-reperfusion injury (18). The expression of HIF-1α is slightly upregulated under hypoxic conditions, which is a protective mechanism against hypoxia (19, 20). The HIF-1/HRE pathway can participate in the myocardial protection of ischemic pre-/post-conditioning by regulating the oxidative respiratory chain complex IV, PI3K-Akt pathway, NO-PKG pathway, and the adenosine pathway (21–23). There has been a lack of research on the mechanism of the HIF-1/HRE pathway in pharmacological pre-/post-conditioning. Therefore, it is interesting to determine whether pharmacological post-conditioning, which confers myocardial protection, is pertinent to the HIF-1/HRE signaling pathway.

To investigate this hypothesis, this experiment used an hypoxia-reoxygenation model of adult rat cardiomyocytes, excluding the influence of nerve-humoral regulation and peripheral vascular status. We examined the protective effect of diazoxide post-conditioning and the underlying mechanism of diazoxide post-conditioning activation of the HIF-1/HRE pathway in hypoxia-reoxygenation cardiomyocytes. Previous studies have revealed that diazoxide increases HIF-1α protein levels to reduce damage afflicted by hypoxia in the pancreatic cells (24). We first examined whether the administration of diazoxide post-conditioning could activate the HIF-1/HRE pathway in hypoxia-reoxygenation cardiomyocytes. The results showed that diazoxide post-conditioning significantly elevates the protein expression levels of HIF-1α, VEGF, and bcl-2, leading to improvements in the myocardial ultrastructure, mitochondrial Flameng score, and membrane potential. In addition, our previous research has shown that inhibition of the HIF-1/HRE pathway by the HIF-1α subunit blocker, 2ME2, can eliminate the myocardial protective effects of diazoxide post-conditioning in isolated rat hearts (25, 26). On this basis, we have confirmed that the myocardial protective effects of diazoxide post-conditioning can be attributed to upregulation of the HIF-1/HRE pathway.

Several studies have shown that the mitoK_ATP_ channel opener, diazoxide, reduces brain and hepatic ischemia-reperfusion injury, with a protective effect that appears to be mediated by the opening of mitoK_ATP_ (27, 28). In animal myocardial ischemia/reperfusion models, activation of mitoK_ATP_ channels by pharmacological openers has been shown to attenuate myocardial infarction and endothelial dysfunction. Conversely, blockage of the mitoK_ATP_ channels aggravates myocardial necrosis and the no-reflow phenomenon after ischemia/reperfusion. Opening of mitoK_ATP_ channels in cultured adult rat cardiomyocytes resulted in significant signal pathway changes, while the AKT-forkhead box (Foxo)1, F1F0-ATPase, and mitogen-activated protein kinase (MAPK) pathways have been shown to be regulated by mitoK_ATP_ opening to protect cardiomyocytes against hypoxia (29–31). We wondered whether the HIF-1/HRE pathway was also regulated by mitoK_ATP_. Our results reveal that after inhibition of mitoK_ATP_ by 5-HD+D, diazoxide was not able to upregulate the expression of HIF-1α, VEGF, or bcl-2, resulting in increased ultrastructure pathology, mitochondrial Flameng score, and inflammatory factors. As revealed in this study, activation of the HIF-1/HRE pathway is also mediated by mitoK_ATP_ channel opening. Therefore, we found that diazoxide post-conditioning can noticeably activate the HIF-1/HRE pathway by opening the mitoK_ATP_ channel.

A growing number of investigations have demonstrated that pharmacological post-conditioning allows opening of the mitoK_ATP_ channels, with the resulting increased K^+^ influx into the matrix causing an increase in ROS (31–33). In addition, diazoxide induces mitochondrial depolarization and stimulates ROS generation via activation of the mitoK_ATP_ channel. Mitochondrial membrane depolarization by diazoxide is reversible and may increase ROS production without inducing apoptosis (34). Previous studies have indicated that localized transient elevation of ROS in the mitochondria is protective against ischemic injury. Diazoxide stimulates vascular repair-relevant functions of CD34+ cells via mitoK_ATP_-dependent ROS release, and the mitoK_ATP_-mediated ROS released in the mitochondria offers cerebral protection from ischemic injury (35). In this study, we found that MPG could reverse the effect of diazoxide post-conditioning, resulting in a block effect on the HIF-1/HRE pathway, suggesting that ROS released by the mitoK_ATP_ channel contribute to activation of the HIF-1/HRE pathway in diazoxide post-conditioning.

In addition, this study found that diazoxide post-conditioning promotes mitoK_ATP_ channel release of ROS to activate the HIF-1/HRE pathway by inhibiting HIF-1α degradation. Mitochondrial ROS is critical to O_2_-sensing in hypoxic cells (36), and an increase in ROS production facilitates the hypoxia-mediated transition of ferrous iron to its ferric state (37). The increase in ferric iron facilitates the rapid inhibition of HIF-1α-prolyl hydroxylase (PHD) activity (38), which regulates levels of HIF-1α protein. We hypothesized that the protective mechanisms of mitochondrial ROS were crucial to stabilization HIF-1α; therefore, DMOG following MPG was used to investigate the role of ROS. DMOG inhibited the prolyl-hydroxylation of the α-subunit catalyzed by PHD, thereby precluding HIF-1α from VHL-mediated degradation. Interestingly, our study found that the effect of an absence of mitochondrial ROS in inhibiting the HIF-1/HRE pathway could be reversed by DMOG, thereby demonstrating that ROS could play a similar role to DMOG. Mitochondrial ROS suppressed PHD-induced HIF-1α degradation. According to HIF-1α, mRNA levels were independent of hypoxic status or pharmacological treatment, suggesting that diazoxide post-conditioning did not impact HIF-1α transcriptional activity, but the accumulation and stability of HIF-1α protein. On this basis, this paper has found that mitochondrial ROS may inhibit canonical PHD-mediated pathways of HIF-1α degradation in diazoxide post-conditioning.

Physiological ROS signaling has an important regulatory role in cellular homeostasis. The spatial and temporal characteristics of ROS formation determine its influence on different cellular organelles that may stimulate the cellular functions or may interfere with cellular homeostasis leading to apoptosis. Previous studies have shown that excessive ROS allows a state of oxidative stress that can precipitate cellular dysfunction (39–42); however, moderate levels of ROS appear to act as a signal transduction factor and second messenger to guide gene regulation in a variety of cell types, essentially regulating downstream signals (43). To further explore the role of mitochondrial ROS in diazoxide post-conditioning and the relationship between changes in concentration and the HIF-1/HRE pathway, this experiment monitored ROS levels at early reoxygenation (25 min) and at the end of reoxygenation (120 min). The results indicated that, in the early stages of reoxygenation after hypoxia, cells can produce excessive ROS instantaneously, exceeding the ability of cells to resist oxidative stress, and resulting in irreversible and severe damage to the cell structure. Diazoxide post-conditioning allows opening of the mitoK_ATP_ channels, producing moderate levels of ROS, which could activate the HIF-1/HRE pathway, upregulate the anti-oxidative stress system of the cell, and maintain cell morphology and function. Moreover, the moderate levels of ROS generated by the opening of the mitoK_ATP_ channel inhibits HIF-1α degradation, meaning that when MPG eliminated ROS, activation of diazoxide post-conditioning on the HIF-1/HRE pathway was decreased. Interestingly, ROS fulfills an important modulatory role of HIF-1α activity in two different ways: under normal circumstances, nicotinamide adenine dinucleotide phosphate (NADPH) oxidase is responsible for producing ROS, which induces the degradation of HIF-1α. Under hypoxia and pharmacological intervention, mitochondrial production of ROS stimulates the stabilization of HIF-1α by modifying the response of PHDs, causing the stable HIF-1α protein (44–46) to bind with HIF-1β to activate the HIF-1/HRE pathway, thereby reducing the oxidative stress damage caused by MIRI.

## Limitations

ROS is the general term for O^2−^, H_2_O_2_, NO, and –OH radicals and other single-electron reduction products, each of which has been proposed as signaling ROS. In this study, the role of every component of ROS was not tested in HIF-1/HRE pathway activation. In addition, the specific concentration range of moderate levels of ROS acting as a second messenger to guide HIF-1/HRE pathway regulation needs further investigation. Furthermore, the effects of HIF-1/HRE pathway activation in diazoxide post-conditioning still need to be explored.

## Conclusion

This study shows that diazoxide post-conditioning promotes the opening of mitoK_ATP_ channels, generating a moderate level of ROS which may inhibit HIF-1α degradation to activate the HIF-1/HRE pathway. This further results in an increase of the anti-oxidation ability of the cell, leading to the stabilization of cardiomyocyte function and structure.

## Data Availability Statement

The original contributions presented in the study are included in the article/supplementary materials, further inquiries can be directed to the corresponding author/s.

## Ethics Statement

The studies involving animals were reviewed and approved by the Affiliated Hospital of Zunyi Medical University, China.

## Author Contributions

X-YC, W-JZ, and TY conceived and designed the experiments. X-YC, J-QW, and S-JC performed the experiments. X-YC, J-QW, YW, and M-YD analyzed the data. X-YC, W-JZ, and H-YW wrote the paper. All authors contributed to the article and approved the submitted version.

## Funding

This work was supported by Master's Research Foundation of the Affiliated Hospital of Zunyi Medical College [Grant No. (2016)49] and the Science and Technology Fund Projects of Guizhou Provincial Health department (Grant No. gzwjkj2019-1-163).

## Conflict of Interest

The authors declare that the research was conducted in the absence of any commercial or financial relationships that could be construed as a potential conflict of interest.

## Publisher's Note

All claims expressed in this article are solely those of the authors and do not necessarily represent those of their affiliated organizations, or those of the publisher, the editors and the reviewers. Any product that may be evaluated in this article, or claim that may be made by its manufacturer, is not guaranteed or endorsed by the publisher.

## References

[1] Kivimäki M Jokela M Nyberg ST Singh-Manoux A Fransson EI Alfredsson L. Long working hours and risk of coronary heart disease and stroke: a systematic review and meta-analysis of published and unpublished data for 603,838 individuals. Lancet. (2015) 386:1739–46. 10.1016/S0140-6736(15)60295-1 26298822

[2] Kim SY Yang EG. Recent advances in developing inhibitors for hypoxia-inducible actor prolyl hydroxylases and their therapeutic implications. Molecules. (2015) 20:20551–68. 10.3390/molecules201119717 26610437 PMC6332328

[3] Adamovich Y Ladeuix B Golik M Koeners MP Asher G. Rhythmic oxygen levels reset circadian clocks through HIF1. Cell Metab. (2017) 25:93–101. 10.1016/j.cmet.2016.09.014 27773695

[4] Zhang Z Yao L Yang J Wang Z Du G. PI3K/Akt and HIF1 signaling pathway in hypoxiaischemia. Mol Med Rep. (2018) 18:3547–54. 10.3892/mmr.2018.9375 30106145 PMC6131612

[5] Cai Z Hua Z Bosch MM Fox TK Wang L Wei C. Complete loss of ischaemic preconditioning-induced cardioprotection in mice with partial deficiency of HIF-1. Cardiovasc Res. (2008) 77:463–70. 10.1093/cvr/cvm035 18006459

[6] Li QF Xu H Sun Y Hu R Jiang H. Induction of inducible nitric oxide synthase by isoflurane post-conditioning *via* hypoxia inducible factor-1α during tolerance against ischemic neuronal injury. Brain Res. (2012) 1451:1–9. 10.1016/j.brainres.2012.02.055 22445062

[7] Costa ADT Jakob R Costa CL Andrukhiv K West IC Garlid KD. The mechanism by which the mitochondrial ATP-sensitive K+ channel opening and H2O2 inhibit the mitochondrial permeability transition. J Biol Chem. (2006) 281:20801–08. 10.1074/jbc.M600959200 16720572

[8] Santos AL Sanchari S Lindner AB. The good, the bad, and the ugly of ROS: new insights on aging and aging-related diseases from eukaryotic and prokaryotic model organisms. Oxid Med Cell Longev. (2018) 2018:1–23. 10.1155/2018/1941285 29743972 PMC5878877

[9] Li X Ni L Wang W Zong L Yao B. LncRNA Fendrr inhibits hypoxia/reoxygenation-induced cardiomyocyte apoptosis by downregulating p53 expression. J Pharm Pharmacol. (2020) 72:1211–20. 10.1111/jphp.13298 32537758

[10] Wang GW Zhou Z Klein JB Kang YJ. Inhibition of hypoxia/reoxygenation-induced apoptosis in metallothionein-overexpressing cardiomyocytes. Am J Physiol Heart Circ Physiol. (2001) 280:H2292–9. 10.1152/ajpheart.2001.280.5.H2292 11299233

[11] Flameng W Borgers M Daenen W Stalpaert G. Ultrastructural and cytochemical correlates of myocardial protection by cardiac hypothermia in man. J Torac Cardiovasc Surg. (1980) 79:413–24. 10.1016/S0022-5223(19)37950-4 6243726

[12] Yellon DM Hausenloy DJ. Myocardial reperfusion injury. N Engl J Med. (2007) 357:1121–35. 10.1056/NEJMra071667 17855673

[13] Prompunt E Sanit J Barrère-Lemaire S Noordali H Madhani M Kumphune S. The cardioprotective effects of secretory leukocyte protease inhibitor against myocardial ischemia/reperfusion injury. Exp Ther Med. (2018) 15:5231–42. 10.3892/etm.2018.6097 29904407 PMC5996700

[14] Li X Liu M Sun R Zeng Y Chen S Zhang P. Protective approaches against myocardial ischemia reperfusion injury. Exp Ther Med. (2016) 12:3823–29. 10.3892/etm.2016.3877 28101167 PMC5228114

[15] Ong SB Hernández-Reséndiz S Crespo-Avilan GE Mukhametshina RT Kwek XY Cabrera-Fuentes HA. Inflammation following acute myocardial infarction: multiple players, dynamic roles, and novel therapeutic opportunities. Pharmacol Ther. (2018) 186:73–87. 10.1016/j.pharmthera.2018.01.001 29330085 PMC5981007

[16] Swirski FK Nahrendorf M. Cardioimmunology: the immune system in cardiac homeostasis and disease. Nat Rev Immunol. (2018) 18:733–44. 10.1038/s41577-018-0065-8 30228378

[17] Sivaraman V Yellon DM. Pharmacologic therapy that simulates conditioning for cardiac ischemic/reperfusion injury. J Cardiovasc Pharmacol Ther. (2014) 19:83–96. 10.1177/1074248413499973 24038018

[18] Eckle T Kohler D Lehmann R El Kasmi K Eltzschig HK. Hypoxia-inducible factor-1 is central to cardioprotection: a new paradigm for ischemic preconditioning. Circulation. (2008) 118:166–75. 10.1161/CIRCULATIONAHA.107.758516 18591435

[19] Sarkar K Cai Z Gupta R Parajuli N Fox-Talbot K Darshan MS. Hypoxia inducible factor 1 transcriptional activity in endothelial cells is required for acute phase cardioprotection induced by ischemic preconditioning. Proc Natl Acad Sci USA. (2012) 109:10504–09. 10.1073/pnas.1208314109 22699503 PMC3387090

[20] Jia Z Lian W Shi H Cao C Han S Wang K. Ischemic postconditioning protects against intestinal ischemia/reperfusion injury *via* the HIF-1α/miR-21 axis. Sci Rep. (2017) 7:16190. 10.1038/s41598-017-16366-6 29170412 PMC5700993

[21] Cadenas S Aragones J Landazuri MO. Mitochondrial reprogramming through cardiac oxygen sensors in ischaemic heart disease. Cardiovasc Res. (2010) 88:219–28. 10.1093/cvr/cvq256 20679415

[22] Bento CF Pereira P. Regulation of hypoxia-inducible factor 1 and the loss of the cellular response to hypoxia in diabetes. Diabetologia. (2011) 54:1946–56. 10.1007/s00125-011-2191-8 21614571

[23] Xie P Yang L Talaiti A Wu J Yu J Yu T. Deferoxamine-activated hypoxia-inducible factor-1 restores cardioprotective effects of sevoflurane postconditioning in diabetic rats. Acta Physiol. (2017) 221:98–114 10.1111/apha.12874 28316125

[24] Ma ZH Moruzzi N Catrina SB Hals I Oberholzer J Grill V. Preconditioning with associated blocking of Ca2+ inflow alleviates hypoxia-induced damage to pancreatic β-cells. PLoS ONE. (2013) 8:e67498. 10.1371/journal.pone.0067498 23935835 PMC3723782

[25] Li J Zhou W Chen WJ Wang HY Zhang Y Yu T. Mechanism of the hypoxia inducible factor 1/hypoxic response element pathway in rat myocardial ischemia/diazoxide post-conditioning. Mol Med Rep. (2020) 21:1527–36. 10.3892/mmr.2020.10966 32016463 PMC7003038

[26] Pan YC Wang Y Shi W Liu Y Cao S Yu T. Mitochondrial proteomics alterations in rat hearts following ischemia/reperfusion and diazoxide post-conditioning. Mol Med Rep. (2021) 23:161. 10.3892/mmr.2020.11800 33355377 PMC7789131

[27] Won U Jiye K Jooyoung L Gi-Won S Gyu SH Eunyoung T. Remote ischemic preconditioning and diazoxide protect from hepatic ischemic reperfusion injury by inhibiting HMGB1-induced TLR4/MyD88/NF-κB Signaling. Int J Mol Sci. (2019) 20:5899. 10.3390/ijms20235899 31771292 PMC6929132

[28] Domoki F Perciaccante JV Veltkamp R Bari F Busija DW. Mitochondrial potassium channel opener diazoxide preserves neuronal-vascular function after cerebral ischemia in newborn pigs. Stroke. (1999) 30:2713–8. 10.1161/01.STR.30.12.2713 10583002

[29] Duan P Wang JX Li Y Wei SQ Su F Zhang SL. Opening of mitoKATP improves cardiac function and inhibits apoptosis *via* the AKT-Foxo1 signaling pathway in diabetic cardiomyopathy. Int J Mol Med. (2018) 42:2709–19. 10.3892/ijmm.2018.3832 30132505 PMC6192784

[30] Gavali JT Carrillo ED García MC Sánchez JA. The mitochondrial K-ATP channel opener diazoxide upregulates STIM1 and Orai1 *via* ROS and the MAPK pathway in adult rat cardiomyocytes. Cell Biosci. (2020) 10:96. 10.1186/s13578-020-00460-w 32817784 PMC7424994

[31] Ala-Rämi A Ylitalo KV Hassinen IE. Ischaemic preconditioning and a mitochondrial KATP channel opener both produce cardioprotection accompanied by F1F0-ATPase inhibition in early ischaemia. Basic Res Cardiol. (2003) 98:250–8. 10.1007/s00395-003-0413-z 12835954

[32] Pain T Yang XM Critz SD Yue Y Nakano A Liu GS. Opening of mitochondrial K(ATP) channels triggers the preconditioned state by generating free radicals. Circ Res. (2000) 87:460–6. 10.1161/01.RES.87.6.460 10988237

[33] Lesnefsky EJ Chen Q Tandler B Hoppel CL. Mitochondrial dysfunction and myocardial ischemia-reperfusion: implications for novel therapies. Annu Rev Pharmacol Toxicol. (2017) 57:535–65. 10.1146/annurev-pharmtox-010715-103335 27860548 PMC11060135

[34] Paggio A Checchetto V Campo A Menabò R Di Marco G Di Lisa F. Identifcation of an ATP-sensitive potassium channel in mitochondria. Nature. (2019) 572:609–13. 10.1038/s41586-019-1498-3 31435016 PMC6726485

[35] Joshi S Jarajapu YPR. Mitochondrial depolarization stimulates vascular repair-relevant functions of CD34^+^ cells *via* reactive oxygen species-induced nitric oxide generation. Br J Pharmacol. (2019) 176:4373–87. 10.1111/bph.14529 30367728 PMC6887676

[36] Simon MC. Mitochondrial reactive oxygen species are required for hypoxic HIF alpha stabilization. Adv Exp Med Biol. (2006) 588:165–70. 10.1007/978-0-387-34817-9_15 17089888

[37] Tayor CT. Mitochondria and cellular oxygen sensing in the HIF pathway. Biochem J (2008) 409:19–26. 10.1042/BJ20071249 18062771

[38] Dang CV Kim JW Gao P Yustein J. The interplay between MYC and HIF in cancer. Nat Rev Cancer. (2008) 8:51–6. 10.1038/nrc2274 18046334

[39] Chouchani ET Pell VR Gaude E Aksentijević D Sundier SY Robb EL. Ischaemic accumulation of succinate controls reperfusion injury through mitochondrial ROS. Nature. (2014) 515:431–5. 10.1038/nature13909 25383517 PMC4255242

[40] Morciano G Giorgi C Bonora M Punzetti S Pavasini R Wieckowski MR. Molecular identity of the mitochondrial permeability transition pore and its role in ischemia-reperfusion injury. J Mol Cell Cardiol. (2015) 78:142–53. 10.1016/j.yjmcc.2014.08.015 25172387

[41] Korge P Calmettes G John SA. Weiss, JN. Reactive oxygen species production induced by pore opening in cardiac mitochondria: the role of complex III. J Biol Chem. (2017) 292:9882–95. 10.1074/jbc.M116.768317 28450391 PMC5473241

[42] Buijs N Oosterink JE Jessup M Schierbeek H Stolz DB Houdijk AP. A new key player in VEGF-dependent angiogenesis in human hepatocellular carcinoma: dimethylarginine dimethylaminohydrolase 1. Angiogenesis. (2017) 20:557–65. 10.1007/s10456-017-9567-4 28741166 PMC5660142

[43] Görlach A Kietzmann T. Superoxide and derived reactive oxygen species in the regulation of hypoxia-inducible factors. Methods Enzymol. (2007) 435:421–46. 10.1016/S0076-6879(07)35022-2 17998067

[44] Requejo-Aguilar R Lopez-Fabuel I Fernandez E Martins LM Almeida A Bolaños JP. PINK1 deficiency sustains cell proliferation by reprogramming glucose metabolism through HIF1. Nat Commun. (2014) 5:4514. 10.1038/ncomms5514 25058378

[45] Belaidi E Morand J Gras E Pépin JL Godin-Ribuot D. Targeting the ROS-HIF1-endothelin axis as a therapeutic approach for the treatment of obstructive sleep apnea-related cardiovascular complications. Pharmacol Ther. (2016) 168:1–11. 10.1016/j.pharmthera.2016.07.010 27492897 PMC5643507

[46] Zepeda AB Pessoa A Jr Castillo RL Figueroa CA Pulgar VM Farías JG. Cellular and molecular mechanisms in the hypoxic tissue:role of HIF-1 and ROS. Cell Biochem Funct. (2013) 31:451–9. 10.1002/cbf.2985 23760768

