# Corrigendum: Diazoxide post-conditioning activates the HIF-1/HRE pathway to induce myocardial protection in hypoxic/reoxygenated cardiomyocytes

**DOI:** 10.3389/fcvm.2023.1281995

**Published:** 2023-09-13

**Authors:** Xi-Yuan Chen, Jia-Qi Wang, Si-Jing Cheng, Yan Wang, Meng-Yuan Deng, Tian Yu, Hai-Ying Wang, Wen-Jing Zhou

**Affiliations:** ^1^ Department of Anesthesiology, The Affiliated Hospital of Zunyi Medical University, Guizhou, China; ^2^ Department of Anesthesiology, The Xinqiao Hospital of Army Medical University, Chongqing, China; ^3^ Guizhou Key Laboratory of Anesthesia and Organ Protection, Affiliated Hospital of Zunyi Medical University, Guizhou, China

**Keywords:** hypoxic reoxygenation injury, diazoxide, myocardial protection, HIF-1/HRE pathway, cardiomyocytes

A Corrigendum on Diazoxide post-conditioning activates the HIF-1/HRE pathway to induce myocardial protection in hypoxic/reoxygenated cardiomyocytes by Chen X-Y, Wang J-Q, Cheng S-J, Wang Y, Deng M-Y, Yu T, Wang H-Y and Zhou W-J (2021). Front. Cardiovasc. Med. 8:711465. doi: 10.3389/fcvm.2021.711465

**Incorrect Funding**

In the published article, there was an error in the Funding statement. “This work was supported by Master's Research Foundation of the Affiliated Hospital of Zunyi Medical College [Grant No. (2016)49] and the Science and Technology Fund Projects of Guizhou Provincial Health department (Grant No. gzwjkj2019-1-162).”

The correct Funding statement appears below.

**FUNDING**

This work was supported by Master's Research Foundation of the Affiliated Hospital of Zunyi Medical College [Grant No. (2016)49] and the Science and Technology Fund Projects of Guizhou Provincial Health department (Grant No. gzwjkj2019-1-163).

The authors apologize for this error and state that this does not change the scientific conclusions of the article in any way. The original article has been updated.

## Publisher's note

All claims expressed in this article are solely those of the authors and do not necessarily represent those of their affiliated organizations, or those of the publisher, the editors and the reviewers. Any product that may be evaluated in this article, or claim that may be made by its manufacturer, is not guaranteed or endorsed by the publisher.

